# Dengue in parts of the Guinea Savannah region of Nigeria and the risk of increased transmission

**DOI:** 10.1093/inthealth/ihaa033

**Published:** 2020-06-20

**Authors:** C A Omatola, A B Onoja, E Moses, M Mahmud, C K Mofolorunsho

**Affiliations:** Department of Microbiology, Kogi State University, Anyigba, Kogi State, Nigeria; Department of Virology, College of Medicine, University of Ibadan, Nigeria; Department of Microbiology, Kogi State University, Anyigba, Kogi State, Nigeria; Department of Microbiology, Kogi State University, Anyigba, Kogi State, Nigeria; Department of Microbiology, Kogi State University, Anyigba, Kogi State, Nigeria

**Keywords:** dengue, IgG, Guinea Savannah, Nigeria

## Abstract

**Background:**

Dengue virus (DENV) is spreading to parts of the world where it had not been previously reported. Increased international travel has led to intercontinental importation of dengue by visitors returning from countries in sub-Saharan Africa. Although dengue is well documented in the rain forest region of Nigeria, there is a dearth of information in the Guinea Savannah region, which is a major transit point for local and international visitors in the most populous nation in Africa. We provide preliminary evidence of dengue activity in the Guinea Savannah and highlight the risk factors.

**Methods:**

Blood was collected from a cross-section of 200 patients attending four hospitals in Anyigba, Kogi State. Anti-dengue antibody was identified using DENV immunoglobulin G (IgG) immunoassays. Questionnaires were used to obtain sociodemographic variables and risk factors. Data were analysed with SPSS version 16.0 for Windows.

**Results:**

Forty-two (20.5%) participants had anti-DENV IgG antibodies. Persons within the 45–59 y age group were more seropositive, with a rate of 35%. Males were more seropositive compared with females. Marriage, formal education, involvement in business activities and the presence of grasses around homes were associated with higher IgG seropositivity. The presence of open water containers around human dwellings and a lack of mosquito net use are predisposing factors.

**Conclusions:**

This study identified past exposure to DENV among people in Anyigba, located in the Guinea Savannah region. Proper diagnosis of febrile episodes is required to improve case management and curtail off-target treatment. The high rate of previous exposure of patients to dengue indicates the need to strengthen vector control and dengue surveillance programs.

## Introduction

Dengue is caused by dengue virus (DENV), which belongs to the family Flaviviridae, genus *flavivirus*. Four serotypes (DENV1–4) have been identified that cause dengue. More than 2.5 billion people reside in dengue-endemic countries around the world, where approximately 390 million people are infected with dengue.^1^ In those countries, about 50–100 million new cases occur annually.^2^ DENV is transmitted by *Aedes aegypti*, which is the principal vector, and *Aedes albopictus*, which is a less competent vector. The vector is spreading across the world as a result of warmer climates, thereby facilitating transmission of dengue to countries where it had not been reported before.^3^ This has increased the burden of dengue and other arboviral diseases.^4,5^

In December 2015, Dengvaxia (CYD-TDV) was licensed in Mexico for use in individuals between 9 and 45 y of age. It is now licensed in 20 dengue-endemic countries,^6^ however, use is limited because of safety concerns and age of administration and seroprevalence requirements.^7^ No specific antiviral drug is currently available for treatment of dengue. As such, management of clinical cases is by the amelioration of symptoms.^8^

The incidence of dengue has risen 30-fold in the past 50 y because of population growth, global warming, inefficient mosquito control, urbanization and poor DENV surveillance.^4^ It is a neglected disease that is causing outbreaks and epidemics in urban and semi-urban areas in developing countries.^9^ High rates of dengue have been reported in the rainforest region of Nigeria.^10^ All DENV serotypes have been identified in the country.^11^ Dengue haemorrhagic fever (DHF) and dengue shock syndrome (DSS) are severe forms of dengue that cause high morbidity and mortality. In recent times, DHF has been reported to be emerging in Nigeria.^12^

Most healthcare workers are unaware of the burden of dengue and, because many laboratories are not equipped for diagnosing the condition, most cases are misdiagnosed as malaria or typhoid fever, leading to mismanagement.^13^ As a result, there is substantial underreporting of dengue within health systems.^14^ Indeed, tropical climates favour the spatial distribution of dengue vectors,^15^ and considering the endemicity of malaria and other arboviruses in the Guinea Savannah region,^16^ there is a need to investigate the occurrence of dengue in this region. Although dengue poses a serious public health problem in the rainforest region of West Africa,^10^ data are unavailable in many parts of the Guinea Savannah. Therefore, because of the heavy human traffic, including foreign visitors in the Guinea Savannah region of Nigeria, it is imperative to investigate the immune status of people in the area to provide baseline data that will inform policy decisions and guide travellers regarding risk factors for dengue in the area.

## Materials and methods

### Sampling locations

Four hospitals in Anyigba were selected for this study because of the large patient enrolment from different parts of Kogi State, Nigeria. The hospitals are Maria Goretti Hospital, Christ the Good Shepherd Hospital, Grimard Catholic Hospital and Amazing Grace Specialist hospital. Anyigba has a tropical climate characterized by an average annual temperature of 37°C and a rainy season from April to October. It is a transit point for road travellers from the northern and southern parts of Nigeria. Poor drainage systems, unkempt bushes around human dwellings and indiscriminate dumping of refuse are evident in the area.

### Study design

This is a cross-sectional study involving 200 visiting outpatients randomly selected from the four hospitals from February to April 2019 in Anyigba. With the assistance of the hospital personnel, numbers were assigned to each patient that met the inclusion criteria and this was written on the patient's card. Thereafter, a random number generator identified the participants. About 16 samples were collected per visit from participants enrolled for malaria and typhoid examination. Informed consent was sought and participants who gave oral or written consent were recruited into the study. Parental assent was obtained for participants who were <15 y of age. With the assistance of hospital personnel, relevant clinical and sociodemographic data were collected using a structured questionnaire at the time of sample collection. Inclusion criteria were fever and suspected malaria or typhoid infection.

### Ethical considerations

Ethical approval for this study was obtained from the Institutional Review Board of Kogi State University in accordance with the ethical code of the World Medical Association Declaration of Helsinki.

### Sample collection, processing and storage

A total of 2 ml of whole blood was collected by venipuncture from each consenting participant in a plain sterile sample tube. Whole blood was spun at 3000 rpm for 5 min to obtain serum, which was stored at −20°C until sampling was completed. A second aliquot of blood was used for malaria parasite examination and tidal assay.

### Assay for DENV immunoglobulin G (IgG) antibody

IgG is produced 11 d after the onset of dengue and can be found in the long term after infection. DENV IgG detection kits were obtained from Biopanda Diagnostics (Belfast, UK). Membrane was pre-coated with mouse anti-human IgG. If antibodies are present in the sera, they react with conjugated recombinant DENV antigen. Chromatographic migration on the membrane occurs by capillary action to react with mouse anti-human IgG, which produces a coloured line that is indicative of a positive result. Another coloured line is expected to validate the assay, indicating that an appropriate specimen volume was added.

### Statistical analysis

Data obtained from this study were analysed using Statistical Package for Social Sciences (version 16.0; IBM, Armonk, NY, USA). Descriptive data are presented in tables. Differences between categorical variables and prevalence rates were determined using the chi-square test. A p-value <0.05 was considered statistically significant.

## Results

In total, 39% males and 61% females participated in this study. The highest enrolment was for people between 15 and 29 y of age (38.5%) followed by those who were 30–44 y (30.5%) (Table 1). The prevalence of anti-DENV IgG antibody was 20.5% (n=42). Patients in 45–59 y age group had the highest seropositivity of 35%. Although antibody was detected among all age groups except those >74 y of age, there was no statistically significant difference between age and IgG seropositivity (p>0.05). There were more females than males in this study (male:female ratio 1:1.6), however, the anti-DENV seropositivity rate was higher in males (23.1%) compared with females (20.5%) (Table 2). Participants with no formal education had higher anti-DENV seropositivity (27.1%) compared with participants with a tertiary education (15.3%). However, this difference was not statistically significant (p>0.05). Higher IgG rates were observed among married couples (28.3%) compared with 23.1%, 15.7% and 0% rates for widows, singles and divorced participants, respectively. Although high seropositivity rates were observed in all occupational groups, participants involved in businesses such as tailoring, trading and apprentices were at higher risk of dengue. Participants who seldom used mosquito nets and those who shared beds had higher rates of 23.2% and 25%, respectively, compared with 18.7% and 16.3%, respectively, among consistent net users and participants who slept alone. The presence of hollow containers, nearby streams or wells and bushes around homes predisposed participants to higher risks of dengue.

**Table 1.** Frequency of sex and age occurrence of febrile patients in Anyigba

		Distribution of participants by study location				
Variable	Participants, n	MGH, n (%)	CGCH, n (%)	GCH, n (%)	AGH, n (%)	Proportion, %
Gender						
Male	78	21 (26.9)	21 (26.9)	16 (20.5)	20 (25.6)	39
Female	122	29 (23.8)	29 (23.8)	34 (27.9)	30 (24.6)	61
Age group (years)						
<15	28	11 (39.3)	0 (0.0)	10 (35.7)	7 (25.0)	14
15–29	77	20 (26.0)	30 (39.0)	15 (19.5)	12 (15.6)	38.5
30–44	61	10 (16.4)	15 (24.6)	13 (21.3)	23 (37.7)	30.5
45–59	20	3 (15.0)	4 (20.0)	9 (45.0)	4 (20.0)	10
60–74	12	6 (50.0)	1 (8.3)	0 (0.0)	2 (16.7)	6.0
>74	2	0 (0.0)	0 (0.0)	0 (0.0)	2 (100)	1.0

There were 50 people per hospital. MGH: Maria Goretti Hospital; CGCH; Christ the Good Shepherd Hospital; GCH: Grimard Catholic Hospital; AGH: Amazing Grace Specialist Hospital.

**Table 2.** Age and sex of febrile patients with IgG in Anyigba, Kogi State

		Anti-DENV antibodies, n (%)					
Variable	Participants, n	MGH	CGCH	GCH	AGH	Prevalence	p-Value
Gender							
Male	78	4 (5.1)	3 (3.8)	5 (6.4)	4 (5.1)	18 (23.1)	
Female	122	8 (6.6)	6 (4.9)	5 (4.1)	6 (4.9)	25 (20.5)	0.66
Total	200	12 (6.0)	9 (4.5)	10 (5.0)	10 (5.0)	43 (21.5)	
Age group (years)							
<15	28	5 (17.9)	0 (0)	0 (0)	1 (3.6)	6 (21.4)	
15–29	77	1 (1.3)	5 (6.5)	1 (1.3)	4 (5.2)	12 (15.6)	
30–44	61	3 (4.9)	4 (6.6)	2 (3.3)	5 (8.2)	15 (24.6)	
45–59	20	2 (10.0)	0 (0)	5 (25.0	0 (0)	7 (35.0)	0.45
60–74	12	1 (8.3)	0 (0)	2 (16.7)	0 (0)	3 (25.0)	
>74	2	0 (0.0)	0 (0)	0 (0)	0 (0)	0 (0)	
Total	200	12 (24)	9 (18.0)	10 (5.0)	10 (5.0)	43 (21.5)	

## Discussion

Most febrile illnesses in Nigeria are treated as malaria or typhoid, without considering possible arboviral aetiologies, which have similar symptoms. As a result, misdiagnosis is common and laboratories do not have effective means of diagnosis.^17^ IgG is produced 11 d after the onset of dengue and this is long lasting. However, when a fourfold or greater value of IgG is detected in acute and convalescent individuals, it shows infection was recent.^18^ Since the aim of this preliminary study was to identify the presence of IgG, we did not quantify the IgG level. In this study, although febrile patients were enrolled (Table 1), 20.5% of participants were who were randomly sampled were previously exposed to dengue. Optimal temperatures and favourable environmental conditions such as the presence of bushes and increased vector breeding facilitate DENV transmission in the area, and this is responsible for the high prevalence in this study. The rate is comparable to the 21.8% reported earlier in the Republic of Djibouti,^19^ although higher rates were reported in Tanzania.^20^ This disparity can be attributed to differences in environmental factors such as rainfall, temperature and humidity, which positively impact DENV transmission.^21^ In this area, males are more involved in agricultural activities and are frequently exposed during periods of intense vector activity (Table 2). This high seropositivity in males is similar to previous reports in Jigawa State, an arid part of Nigeria.^22^ High anti-DENV seropositivity was observed in all age groups, except participants >75 y of age, reinforcing the endemicity of dengue in the area. Although there was no significant difference between the age of the peak IgG level (45–59 y) (Table 2) and anti-DENV seropositivity, an increase in age is associated with a greater possibility of exposure to dengue. This age group constitutes the active workforce, who are more outgoing and engage in various occupations and recreational activities.

Educational attainment of participants was positively correlated to DENV seropositivity in this study. Those with lower education levels had higher exposure to dengue (Table 3). This is because participants with higher education levels are better informed about effective methods of prevention and control. High dengue prevalence among married participants in this study may be because couples maintain close contact compared with widows or widowers, thus if one partner is infected, the other can be horizontally infected. Participants engaged in businesses such as tailoring, selling, hair cutting and farming were more predisposed to dengue compared with other occupational groups (Table 3). Similarly, high seroprevalence was reported among farmers in northwest Ethiopia.^23^ Farming and the other aforementioned businesses are done outdoors during the day, and this increases the chances of exposure to *Aedes* sp. Patients with grasses, open water containers or streams or wells around their homes had higher dengue IgG levels (Table 3). These factors provide favourable breeding ground for vectors, causing increased vector–human interactions and dengue transmission. Participants who shared a bed were more seropositive for IgG antibodies compared with those who slept alone, as sharing a bed increases feeding opportunities for mosquitoes. This finding is consistent with previous reports by Ndosi et al.^24^

**Table 3.** Distribution of IgG antibodies and sociodemographic variables of participants

Variables	Patients tested, n	Positive, n (%)	p-Value
Education level			
None	48	13 (27.1)	
Primary	31	6 (19.4)	0.46
Secondary	62	15 (24.2)	
Tertiary	59	9 (15.3)	
Marital status			
Married	92	26 (28.3)	
Divorced	6	0 (0)	0.12
Widowed/separated	13	3 (23.1)	
Single	89	14 (15.7)	
Occupation			
Farming	29	7 (24.1)	
Civil servant	28	5 (17.9)	0.09
Business	60	19 (31.7)	
Student	83	12 (14.5)	
Use of mosquito net			
Yes	75	14 (18.7)	0.45
No	125	29 (23.2)	
Use of insecticide			
Yes	64	17 (26.6)	0.23
No	136	26 (19.1)	
Number per bed			
1	80	13 (16.3)	0.14
≥2	120	30 (25.0)	
Livestock ownership			
Yes	69	20 (29.0)	0.05
No	131	23 (17.6)	
Uncovered containers around home			
Yes	110	25 (22.7)	0.64
No	90	18 (20.0)	
Used tires around home			
Yes	45	8 (17.8)	0.49
No	155	35 (22.6)	
Stream near home			
Yes	58	14 (24.1)	0.56
No	142	29 (20.4)	
Grasses around home			
Yes	182	42 (23.1)	0.08
No	18	1 (5.6)	

In conclusion, findings from this study show that there is dengue activity in Kogi State despite the fact that it is located within the Guinea Savannah region, which is a buffer vegetation zone that separates the arid northern climate from the rainforest region of southern Nigeria. Findings from this study suggest the need to adopt prevention and control strategies targeted at reducing uncovered water storage containers, both indoors and outdoors, clearing of bushes and covering of wells around homes. It reinforces the need for the Nigerian government to meet the sixth United Nation's Social Development Goal, which aims to ensure the availability and sustainable management of water and sanitation for all.
